# PREdator: a python based GUI for data analysis, evaluation and fitting

**DOI:** 10.1186/1751-0473-9-21

**Published:** 2014-09-24

**Authors:** Christoph Wiedemann, Peter Bellstedt, Matthias Görlach

**Affiliations:** 1 RG Biomolecular NMR Spectroscopy at the Leibniz Institute for Age Research - Fritz Lipmann Institute, Beutenbergstr. 11, 07745 Jena, Germany

**Keywords:** Python3, Matplotlib, Paramagnetic relaxation enhancement, Data fitting

## Abstract

The analysis of a series of experimental data is an essential procedure in virtually every field of research. The information contained in the data is extracted by fitting the experimental data to a mathematical model. The type of the mathematical model (linear, exponential, logarithmic, etc.) reflects the physical laws that underlie the experimental data. Here, we aim to provide a readily accessible, user-friendly python script for data analysis, evaluation and fitting. PREdator is presented at the example of NMR paramagnetic relaxation enhancement analysis.

## Introduction

In nearly all fields of physical, chemical or biological research it is requiered to convert experimental data into mathematical expressions. Particularly the determination of a "best fit" for a series of data points to a mathematical model is a pivotal and potentially time consuming step in the extraction of results and data evaluation.

Nuclear magnetic resonance (NMR) spectroscopy not only provides structural information at the atomic scale on biological macromolecules but also on their dynamics, and hence, a more complete description of the system under investigation. Furthermore, dynamics parameters may contribute also to the understanding of proteins and their interaction with other proteins, nucleic acids or small ligands. The determination of longitudinal (R _1_) or transverse (R _2_) relaxation rates of protons in biological macromolecules deliver valuable molecular dynamics information on the system under investigation. For example, this information can be used to determine the interaction interface between individual domains or subunits on the basis of surface accessibility studies in situations where no other NMR parameters, *e.g.* nuclear Overhauser enhancement (NOE) or chemical shift perturbation data, are observable. In such cases, surface accessibility studies can be performed by using of chemically inert paramagnetic probes, *e.g.* paramagnetic metals, oxygen or nitroxides as cosolvents [1]. Protein residues located in the interior of proteins or at the interaction interface are shielded from the paramagnetic agent and experience a weak paramagnetic relaxation enhancement (PRE). In contrast, residues located at the solvent accessible surface experience a strong PRE.

PRE can experimentally be derived from longitudinal (R _1_) or transverse (R _2_) relaxation rate measurements. A sensitive and reliable measure of transverse PREs can be obtained from cross-peak intensities for the state with and without the paramagnetic cosolvent. Relaxation rates are measured by a series of 2D saturation-recovery spectra (^1^H, ^13^C-HMBC or ^1^H, ^15^N-CRINEPT [2]), in which the time delay during which relaxation takes place is gradually increased. The experiments are repeated with different concentrations of the paramagnetic agent. To extract the relaxation rates the signal intensities are fitted to $I=I_{0}(1-e^{-R_{i}t})$ where I _0_ is the intensity after infinite recovery delay, R _i_ is the longitudinal or transverse relaxation rate and t is the time. The PRE is calculated and is represented by the slope of the relaxation rate as a function of the concentration of the paramagnetic agent [3-5].

Even though, a variety of tools (*e.g.* MATLAB 8.0 and Statistics Toolbox 8.1 (The MathWorks, Inc., Natick, MA, US), GNU Octave [6] or R [7]) and NMR-software suites (NMRView [8], CCPN [9], ROTDIF [10]) are available for the extraction and fitting of relaxation data, here we provide a straightforward Python3 based application with a graphical user interface not only for the extraction of relaxation data but also for the calculation of PREs. However, the script should also be useful for fitting and evaluation of virtually any set of data series.

## Implementations and results

PREdator was initially conceived for the analysis of PRE. The application was written in a Mac OS X environment, but it can be run under any operating system for which a Python3 interpreter is available. Python3 and the packages Matplotlib [11], SciPy/NumPy [12] and dill [13] are required to run PREdator.py. Matplotlib [11] is used for data visualisation. All generated plots can be saved as either raster (PNG) or vector format files (PDF or EPS). PREdator also provides the option to save the current session and to restore it later. For data serialization the dill package is implemented [13].

The input file has to contain comma separated data (see example files provided with the download package).

In an initial dialogue the user has the opportunity to choose a predefined fitting function from a list or to enter a self-defined fitting function with up to three fitting parameters. The implementation of NumPy allows to create self-defined fitting functions with predefined mathematical expressions (*e.g.* sin, cos or tan).

**Figure 1:**
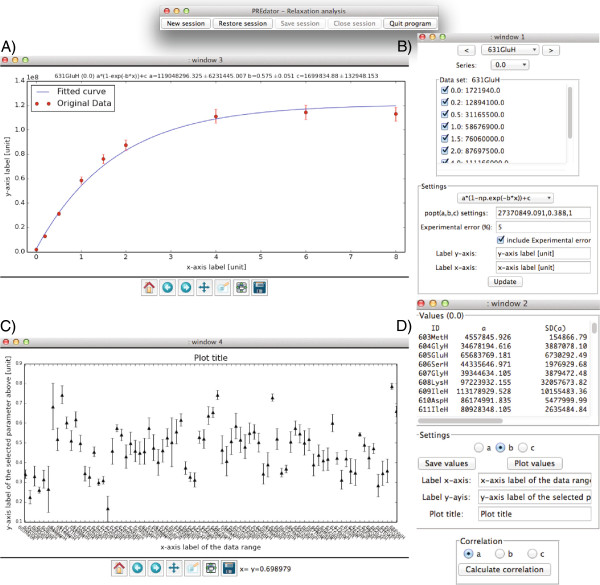
**PREdator interface elements.** The four interactive analysis windows in PREdator. **A)** Graphical representation of the original data and the fitted curve. **B)** Window for selection or deselection of data points that are considered for fitting, drop down menu for fitting function selection, fields for entering initial fitting parameters and experimental error, and entry fields to enter the axis labels for **A)**. **C)** Graphical summary of one of the selectable fitting parameters over the range of submitted data (*e.g.* amino acid residues). **D)** Summary of the resulting fitting parameters, which can be saved as a text file, is shown in the upper text field. The fitting parameter (a,b or c) selected here is displayed over the data range in **C)**. Entry fields to adjust the title and the axis labels for **C)** are also provided.

PREdator provides an initial estimate of the parameters to be fitted. If the user has knowledge of the order of magnitude of the fitting parameters and the experimental error then there is the possibility to enter such initial fitting and error parameters. Data fitting is performed with the curve-fit function implemented in the SciPy-package (modul: scipy.otimize) [12]. For visual inspection the fitted curve and the original data points are shown as graph (Figure 1).

An operating window is provided to re-adjust the fitting function and/or the fitting parameters. Obvious data outliers can be deselected so that they are not considered for fitting. The change of the fitting outcome in the context of the selection and deselection of data points gives a qualitive estimate of fitting robustness. A summary of the fitting results and errors is given in a second window. Fitting errors are provided as one standard deviation errors. The user has the option to save the results to a text file.

For the calculation of the residue-specific PRE, the relaxation rate (R _1_ orR _2_) for each residue and each concentration of the paramagnetic cosolvent are obtained. The cosolvent concentration dependent relaxation rates for individual residues are subsequently correlated by a second fitting. The slope of the resulting fitted function of this second fitting step delivers the PRE for each individual residue.

PREdator delivers fitting parameters in a first step (*e.g.* R _1_ of an individual residue of a protein for different concentrations of the paramagnetic cosolvent). In addition it allows to correlate those fitting parameters, obtained for different conditions, in a second step. The principle of such analysis is not restricted to the evaluation of PREs and is applicable to all kinds of experimental data sets where one type of measurement is repeated under different conditions. Examples include the analysis of fluorescence recovery after photobleaching (FRAP) in a living cell as function of the temperature or the assessment of a DNA-protein interaction under different salt, pH or temperature conditions and to compute properly fitted binding curves. The binding curves in turn can be used to derive the condition-dependent affinity parameter K _*d*_ (equilibrium dissociation constant).

## Conclusions

In summary, PREdator is a time saving tool for visual inspection, fitting and analysis of series of data points. The application is freely accessible at http://nmr.fli-leibniz.de/nmrsoftware.shtml and can be adapted to user requirements.

## Availability and requirements

**Project name:** PREdator**Project homepage:** http://nmr.fli-leibniz.de/nmrsoftware.shtml**Direct Download link:** http://nmr.fli-leibniz.de/PREdator/PREdator.zip**Operating systems:** Linux, Mac OS X and Windows**Programming language:** Python3**Other requirements:** Matplotlib, SciPy/ NumPy, dill**License:** GNU GPL v3**Any restrictions to use by non-academic users:** no licenses are required

## Competing interests

The authors declare that they have no competing interests.

## Authors’ contributions

MG is the principal investigator of the project. CW conceived the idea, prepared the NMR samples, recorded and analyzed the NMR spectra. CW and PB programmed the PREdator script. All authors wrote, read and approved the final manuscript.
